# A viscoelastic–stochastic model of the effects of cytoskeleton remodelling on cell adhesion

**DOI:** 10.1098/rsos.160539

**Published:** 2016-10-19

**Authors:** Long Li, Wenyan Zhang, Jizeng Wang

**Affiliations:** Key Laboratory of Mechanics on Disaster and Environment in Western China, Ministry of Education, College of Civil Engineering and Mechanics, Lanzhou University, Lanzhou, Gansu 730000, People's Republic of China

**Keywords:** cell adhesion, viscoelastic–stochastic model, Monte Carlo simulation

## Abstract

Cells can adapt their mechanical properties through cytoskeleton remodelling in response to external stimuli when the cells adhere to the extracellular matrix (ECM). Many studies have investigated the effects of cell and ECM elasticity on cell adhesion. However, experiments determined that cells are viscoelastic and exhibiting stress relaxation, and the mechanism behind the effect of cellular viscoelasticity on the cell adhesion behaviour remains unclear. Therefore, we propose a theoretical model of a cluster of ligand–receptor bonds between two dissimilar viscoelastic media subjected to an applied tensile load. In this model, the distribution of interfacial traction is assumed to follow classical continuum viscoelastic equations, whereas the rupture and rebinding of individual molecular bonds are governed by stochastic equations. On the basis of this model, we determined that viscosity can significantly increase the lifetime, stability and dynamic strength of the adhesion cluster of molecular bonds, because deformation relaxation attributed to the viscoelastic property can increase the rebinding probability of each open bond and reduce the stress concentration in the adhesion area.

## 1. Introduction

As a salient characteristic in biological systems, cell adhesion plays a pivotal role in cell differentiation, migration and growth [1–7]. Moreover, cell adhesion is crucial in the development and maintenance of tissues [8]. Changes in cell adhesion are associated with many diseases [8,9], such as arthritis [10], cancer [11], osteoporosis [12] and atherosclerosis [13]. Generally, most animal cells survive by adhering to the extracellular matrix (ECM) or other cells [14]. Once the cells have detached from the adhesive substrate, apoptosis may be triggered with the dissociation of receptor–ligand bonds, eventually leading to cell death [15]. Thus, understanding the underlying mechanism of cell adhesion in response to external stimuli is important to advance our fundamental biological and pathological recognitions [16].

During cell adhesion, cells adhere to the ECM through discrete domains of complex multi-molecular assemblies called focal adhesions (FAs), which function like mechanical linkages between the actin stress fibres and the surface of ECM [14,17–20]. The interactions between the cell and ECM via FAs depend on specific molecular bonding, which differs from conventional inter-surface adhesion such as the van der Waals interactions in gecko adhesion [21,22]. In particular, each FA consists of many bonds reversibly formed by specific membrane-bound receptors and their ligands on the surface of ECM. Some studies revealed the regulation effect of ECM stiffness on the mechanical and morphological behaviour of cells via FAs. For example, Engler *et al*. [23] demonstrated that stem cells can be differentiated into different cell lineages depending on the stiffness of substrate. Solon *et al*. [24] found that fibroblasts adapted their internal stiffness to match that of the substrate by aggregating the cytoskeleton and producing internal stresses. Furthermore, experiments demonstrated that cell movement from a soft region to a stiff region can also be guided by the stiffness gradient of the substrate [1]. A logical hypothesis to explain this directional cell locomotion is that cytoskeletal contractility and membrane tension increased when cells adhere to stiff substrate [25,26]. All this research confirmed that cells can perceive the mechanical properties of their environments and generate a response by cytoskeleton remodelling during cell adhesion [27].

In perceiving the mechanical properties of the environment, cells need to exert force at the FAs [28,29]. Generally, these forces can originate from physical interactions or cells' own contractile machinery because stress fibres keep pulling the FAs inwards. Experimental results indicated that the sizes of mature FAs can reversibly increase or decrease when forces are exerted on FAs [30–32], resulting in a constant stress of approximately 5.5 kPa at the adhesion site [31,32].

A number of theoretical models were proposed to elucidate the underlying mechanism of the molecular bond-mediated cell adhesion under various mechanical stimuli. Regarding the reaction kinetics of molecular bonds, Bell [33] and Bell *et al*. [34] established a pioneering theoretical framework to describe the thermodynamic competition between two opposing mechanisms: attractive forces because of ligand–receptor interaction and repulsive forces attributed to electrostatic and osmotic interactions in the glycocalyx layer on the surface of the membrane, leading to bond reformation or breaking. From a statistical point of view, a single molecular bond shows a finite lifetime [33,35–37]. Erdmann & Schwarz [38,39] studied the stochastic evolution of a cluster of molecular bonds under a constant force based on the one-step master equation and suggested that the lifetime of a bond cluster increases with cluster size. Recently, Ju *et al*. [40] investigated the two-dimensional receptor–ligand association kinetics, which was found to be transport regulated. By recognizing that the mechanical properties of the substrate are crucial in the formation and growth of FAs, Gao and his co-workers successfully developed a coupled stochastic–elastic model [41–44] to present insights into the biophysical behaviour in cell adhesion, such as stiffness and orientation-dependent lifetime of cluster bonds [42,45–49], stable size of the molecular bond cluster for cell adhesion [41,45,50], shape-dependent strength of cell adhesion [51–53], enhancement of cell adhesion via pre-tension in the cytoskeleton [54,55] and cell adhesion under cyclic tension [56].

Despite tremendous progress in the study of the cell adhesion problem, most of the aforementioned studies are based on the assumption of purely elastic ECM or cells and focused on the regulations of ECM elasticity in cell adhesion. From the physics point of view, cells are typically viscoelastic materials and the deformation of cells depends on loading history [57], which has been investigated extensively with a variety of quantitative experimental methods, including atomic force microscopy [58], magnetic bead micro-rheometry [59], magnetic micro-needle [60], traction force microscopy [61] and optical tweezers [62]. These experiments highlighted that the local cellular mechanical properties exhibit distinct viscoelasticity, which can be generally regarded as Kelvin-type material with the viscosity in the range from tens to thousands of Pa s. By developing a computational model and performing experiments, Chaudhuri *et al*. [63] determined that the extent of cells cultured on viscoelastic substrates is greater than that of cells cultured on elastic substrates with the same Young's modulus but similar to that of cells cultured on stiffer elastic substrates. These results indicated that substrate viscosity equally contributes in regulating cellular adhesion as substrate elasticity. Considering the adhesion of a spherical membrane containing viscous fluid via molecular bonds and neglecting the effect of cytoskeleton deformation, Gupta [64,65] investigated the effects of fluid viscoelasticity on adhesion strength by assuming multiple- and single-bond adhesion states. Whereas, many results predicted by the proposed models [64,65] are difficult to understand. For example, the conclusion based on the assumption of multiple-bond adhesion under dynamic loading demonstrates that cellular viscoelasticity enhances bond lifetime [64], whereas this outcome does not occur based on the assumption of single-bond adhesion [65] under a constant force, implying that whether or not the viscoelasticity affects bond lifetime seems to depend on the bond number [64,65]. However, this conclusion lacks sufficient biophysical supports.

In this paper, we attempt to address the above problem by proposing a coupled viscoelastic–stochastic model of the cell with viscoelastic properties adhering to an elastic substrate under an external applied force, considering that the mechanism of the effect of cellular viscoelasticity on adhesion behaviour remains unclear. On the basis of this model, we studied the coupled effect of cellular deformation, stress/displacement relaxation, stochastically reforming and breaking of receptor–ligand bonds on adhesion strength and stability.

## 2. Model

**Figure 1.**
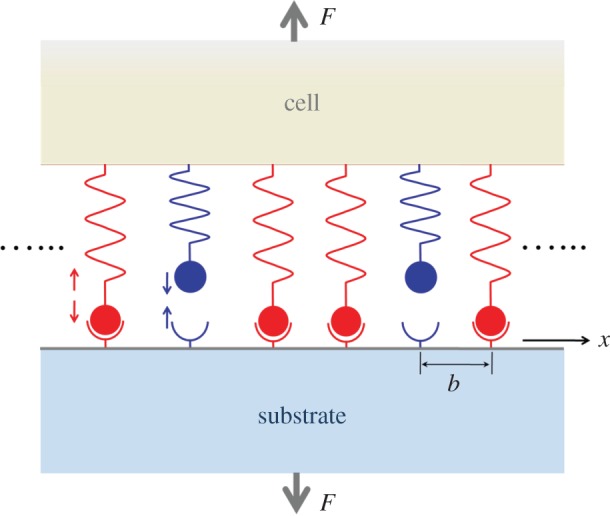
Schematic of the cell–substrate adhesion system. An idealized theoretical model of adhesion between elastic and viscoelastic half spaces through a cluster of receptor–ligand bonds.

To illustrate the viscoelastic–stochastic adhesion model in this study, we consider the plane strain problem of a linear elastic half space with Young's modulus *E*_s_ and a linear viscoelastic Kelvin–Voigt half space with Young's modulus *E*_c_ and viscosity coefficient *η*_c_ linked by a cluster of ligand–receptor bonds under a remotely applied force *F*, as shown in figure 1. In addition, the *x*-axis is placed along the interface between the two half spaces, and the receptor–ligand bonds are uniformly distributed along the *x*-axis, forming an adhesion patch of size 2*a* with bond spacing *b* along both the *x-*axis and the out-of-plane directions, which corresponds to a number density of bonds *ρ*_LR_ = 1/*b*^2^. Consider the closed receptor–ligand bond as a linear spring with stiffness *k*_LR_, rest length *l*_b_ and reacting radius *l*_bind_ around the binding site.

### 2.1. Stress relaxation-dependent interfacial traction

To describe the stress distribution within the adhesion area, we consider the number density of closed bonds at position *x*, and time *t* as *ρ*_LR_*ρ*(*x*, *t*), where *ρ*(*x*, *t*) is the normalized bond density. Thus, the interfacial stress can be given by

**Figure 2.**
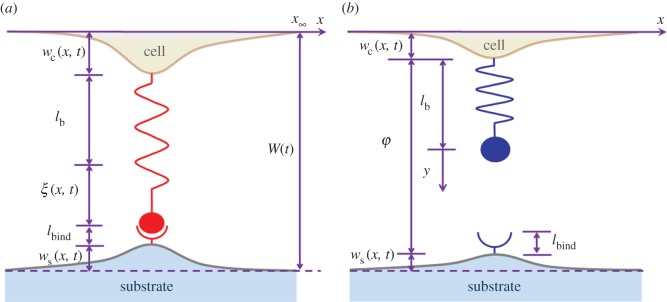
Schematic of the geometrical relationships at position of (*a*) closed bond and (*b*) breaking bond. *W*(*t*) is the surface separation at infinity.

2.1 $$\gamma (x,t)=k_{\mathrm{LR}}\rho_{\mathrm{LR}}\xi (x,t)\rho (x,t),$$

where *ξ*(*x*, *t*) is the elastic extension of the bond at position *x* and time *t*. If we define the relative normal surface deformation as the sum of surface displacements of two half spaces at the closed-bond position *x*, *w*(*x*, *t*) = *w*_c_(*x*, *t*) + *w*_s_(*x*, *t*), as shown in figure 2, we should have

2.2 $$w(x,t)+\xi (x,t)+l_{b}+l_{\mathrm{bind}}=W(t),$$

where subscripts ‘c’ and ‘s’ denote cell and substrate, respectively, and *W* is the interfacial separation between the opposing half spaces at infinity. If the bond at position *x* and time *t* is open, then as shown in figure 2, *φ*(*x*, *t*) = *W*(*t*) − *w*(*x*, *t*) becomes the surface separation at this location.

According to theory of continuum mechanics, the relative normal surface deformation can be related to the interfacial stress as (see appendix A for details)

2.3 $$\frac{\partial w(x,t)}{\partial x}=-\frac{1}{\pi }\int_{-a}^{a}\frac{1}{x-z}\int_{0}^{t}J(t-\tau )\frac{d\gamma (z,\tau )}{d\tau }\;d\tau \;dz,$$

where *J*(*t*) = *J*_s_ + *J*_c_ = 3/(2*E*_s_) + 1/*E*_c_[1 − exp(−*t*/*T*_1_)] denotes the combined creep compliance with a characteristic time scale of *T*_1_ = *η*_c_/*E*_c_.

Applying Laplace transform to equation (2.3) with respect to time *t* yields

2.4 $$\frac{\partial \hat{\bar{w}}(\bar{x},s)}{\partial \bar{x}}=-\frac{\alpha }{\pi }\int_{-1}^{1}\frac{\hat{\bar{\gamma }}(\bar{z},s)}{\bar{x}-\bar{z}}\;d\bar{z},$$

where $\bar{x}=x/a$, $\bar{z}=z/a$, $\bar{w}=w/a$, $\bar{\gamma }(\bar{z},t)=\gamma (\bar{z},t)/ak_{\mathrm{LR}}\rho_{\mathrm{LR}}$ and $\hat{\bar{\gamma }}(\bar{z},s)$ is the Laplace transform of $\bar{\gamma }(\bar{z},t)$,

2.5 $$\alpha =\frac{a\rho_{\mathrm{LR}}k_{\mathrm{LR}}}{\left(E_{s}+E_{c}\right)}s\hat{\bar{J}}(s)$$

and

$$\hat{\bar{J}}(s)=(E_{s}+E_{c})\left(\frac{3}{2E_{s}s}+\frac{1}{E_{c}s}-\frac{1}{E_{c}(E_{c}/\eta_{c}+s)}\right),$$

is the Laplace transform of the normalized combined creep compliance, $\bar{J}(t)=(E_{s}+E_{c})J(t)$.

According to Qian *et al*. [45], *α* in equation (2.5) can be identified as a dynamic stress concentration index to control the stress–relaxation-dependent distribution of interfacial stress. As the integral-differential equation (2.4) has a similar form as the counterpart by Qian *et al*. [45] derived based on elastic theory, thus, similar to the adhesion system of elastic bodies, for the adhesion of viscoelastic medium, a transition from uniform distribution to singular distribution also occurs on the interfacial traction in the adhesion domain when the factor α changes from zero to infinity. For example, when *α* → 0, from equation (2.4) we can derive $w(\bar{x},t)\approx f(t)$, which means that $w(\bar{x},t)$ is a constant at any time *t*, not a function of spatial coordinates. If we further assume that the bonds are uniformly distributed, then the interfacial traction also becomes constant, corresponding to the state of equally shared loading. However, when *α* → ∞, solution of equation (2.4) implies $\gamma (x,t)\propto 1/\sqrt{1-x^{2}/a^{2}}$, which is singular at two edges of the adhesion patch at any time *t*.

On the other hand, considering limiting cases in terms of viscosity, *α* in equation (2.5) can be rewritten as $\alpha_{0}=a\rho_{\mathrm{LR}}k_{\mathrm{LR}}[3/(2E_{s})+1/E_{c}]$, for *η*_c_ → 0, and $\alpha_{\infty }=3a\rho_{\mathrm{LR}}k_{\mathrm{LR}}/(2E_{s})$, for *η*_c_ → ∞, which are linearly proportional to the adhesion size, bond stiffness and density, and inversely proportional to the combined elastic modulus of the cell and substrate. The existence of *α*_∞_ < *α*_0_ indicates that increased viscosity can reduce the stress concentration in the adhesion domain. If we consider the ratio *α*_0_/*α*_∞_ = 1 + 2*E*_s_/(3*E*_c_), we can find that viscosity can reduce stress concentration index *E*_s_/*E*_c_ times. Even if Young's modulus of the substrate becomes similar to the cell, there is still *α*_0_/*α*_∞_ ∼ 1.67. This fact indicates that both the cellular elasticity and viscosity are crucial features used by cells to mediate their biological behaviour.

### 2.2. Stochastic dynamics of single receptor–ligand bonds

A closed single receptor–ligand bond shows a finite lifetime only, which inevitably undergoes a transition from the initial closed state to an open state as a result of thermally activated bond dissociation, even in the absence of an external force. Particularly, the dissociation rate of a closed bond located at *x_n_* is assumed to increase exponentially with the force *F_n_* acting on the bond as [33]

2.6 $$k_{\mathrm{off}}=k_{0}\exp\left(\frac{F_{n}}{F_{b}}\right),$$

where *k*_0_ is the spontaneous dissociation rate in the absence of force and *F*_b_ is a force scale typically in the pN range; 1/*k*_0_ is suggested to be in the range from a fraction of a second to 100s for receptor–ligand bonds in cell adhesion [66,67]. Additionally, the applied force on the closed bond at position *x_n_* can be obtained by *F_n_* = *k*_LR_*ξ*(*x_n_*,*t*).

For free receptor and ligand molecules, bond reform would occur at binding site if the receptor comes sufficiently close to its complementary ligand to react. The bond association rate is assumed to decrease with the separation *φ* between two half spaces as [45,68,69]

2.7 $$k_{\mathrm{on}}=k_{\mathrm{on}}^{0}\frac{l_{\mathrm{bind}}}{Z}\exp\left(-\frac{k_{\mathrm{LR}}{\left(\varphi -l_{b}\right)}^{2}}{2k_{B}T}\right),$$

where $k_{\mathrm{on}}^{0}$ is the spontaneous reaction rate between the receptor and ligand, *k*_B_ is the Boltzmann constant, *T* is the absolute temperature, *l*_bind_ is the reacting radius around the binding site and *Z* is the partition function for a receptor confined in a truncated harmonic potential as shown in appendix B.

It can be seen from equations (2.6) and (2.7) that the bond reaction rates are governed by the forces acting on the closed bond and the surface separation for the open bond, respectively. These force and surface separation can be determined through theory of continuum mechanics as shown in appendix C.

### 2.3. Stochastic–viscoelastic coupling through Monte Carlo simulation

By coupling the deformation of the cell and substrate and stochastic dynamics of molecular bonds, we investigate the spatial and temporal evolutions of receptor–ligand bonds using the ‘first reaction method’ derived from the Gillespie algorithm [70,71]. The key idea of such a method is to sample random trajectories of the system. Specifically, for the adhesion system as shown in figure 1 with total *N* bonds, the detailed simulation process is described as follows:

(1) At time step 0, i.e. $\bar{t}=0$ where $\bar{t}=k_{0}t$ is the normalized time, we set all *N* bonds closed. For a given force *F*, unknown acting forces *F_m_* at each binding site *x_m_*, *m* = 1, 2, … ,*N*, can be obtained based on the force balance relation and the associated force–deformation expression for viscoelastic media, as derived in appendix C.
(2) Calculate the reaction times at individual bond locations *x_m_* by [70,71]
  2.8 $${\bar{t}}_{m}=-\ln\left(\frac{\zeta_{m}}{\mu_{m}}\right),$$
  where *ξ_m_* are the generated series of independent random numbers for individual reaction sites, which are uniformly distributed over the interval [0, 1], *µ_m_* is the normalized reaction rate depending on the bond states at location *x_m_* as *µ_m_* = *k*_off_/*k*_0_ for the closed state and *µ_m_* = *k*_on_/*k*_0_ for the open state. Determine the shortest time $d\bar{t}=\min({\bar{t}}_{m})$ and the corresponding bond location from equation (2.8), for the next bond reaction. The bond state will be updated to open if the bond is currently closed and to closed if it is open. Set lifetime as $\bar{t}=\bar{t}+d\bar{t}$.
(3) Based on the force balance equation and the associated force–deformation relationship for viscoelastic media, we derive the force exerted on each closed bond and the surface separation at each site of open bond, as shown in appendix C.
(4) Proceed to step 2 until all bonds are open.

## 3. Results and discussions

In this study, bond spacing *b* is 32 nm [45], stiffness of each receptor–ligand bond *k*_LR_ is 0.25 pN nm^−1^ [68], rest length of each receptor–ligand bond is 25 nm [56,72], force scale *F*_b_ is 4 pN [38,39], and *a*_0_ = 5 nm denotes the radius of individual bonds [73]. The spontaneous dissociation and association rates are taken as *k*_0_ = 5 s^−1^ [43] and $k_{\mathrm{on}}^{0}=500\;s^{-1}$ [66], respectively. The reaction radius of the receptors and ligands is set to 1 nm [45]. Young's modulus of the substrate and cell is 150 kPa and 10 kPa, respectively.

The relevant parameters adopted in this study are summarized in table 1.

**Table 1.** Parameters in the model.

parameters	value	references
spacing between neighbouring bonds, *b* (nm)	32	[45]
stiffness of receptor–ligand bond, *k*_LR_ (pN nm^−1^)	0.25	[68]
rest length of receptor–ligand bond, *l*_b_ (nm)	25	[56,72]
force scale in bond dissociation, *F*_b_ (pN)	4	[38,39]
radius of individual bonds, *a*_0_ (nm)	5	[73]
spontaneous dissociation rate, *k*_0_ (s^−1^)	5	[43]
spontaneous association rate, $k_{\mathrm{on}}^{0}$ (s^−1^)	500	[66]
reaction radius of receptor and ligand, *l*_bind_ (nm)	1	[45]

### 3.1. Influence of cellular viscosity on the lifetime of receptor–ligand bond cluster

**Figure 3.**
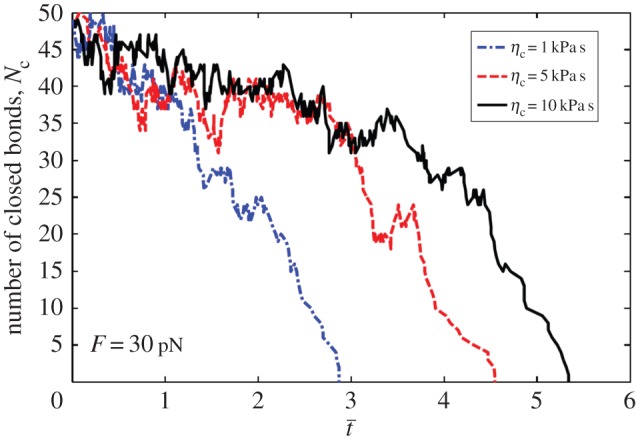
Number of closed bonds as a function of the normalized time under constant force of *F* = 30 pN for total bonds *N* = 50 and different cell viscosity coefficient *η*_c_ = 1, 5 and 10 kPa s.

Figure 3 plots the numerically determined number of closed bonds *N*_c_ as a function of normalized time under a constant external force *F* = 30 pN, and various coefficients of viscosity of the viscoelastic body mimicking the cell: *η*_c_ = 1, 5 and 10 kPa s. It can be seen from figure 3 that a longer lifetime of the cluster of molecular bonds is associated with a larger coefficient of viscosity of the cell.

Why does the cluster lifetime increase with the coefficient of viscosity? Two reasons may cause this phenomenon: first, existence of viscosity can reduce the dynamic stress concentration index as shown in equation (2.5), resulting in much uniformly distributed interfacial stress; second, the creep effect of the viscoelastic material can regulate the rebinding rate of each open bond.

**Figure 4.**
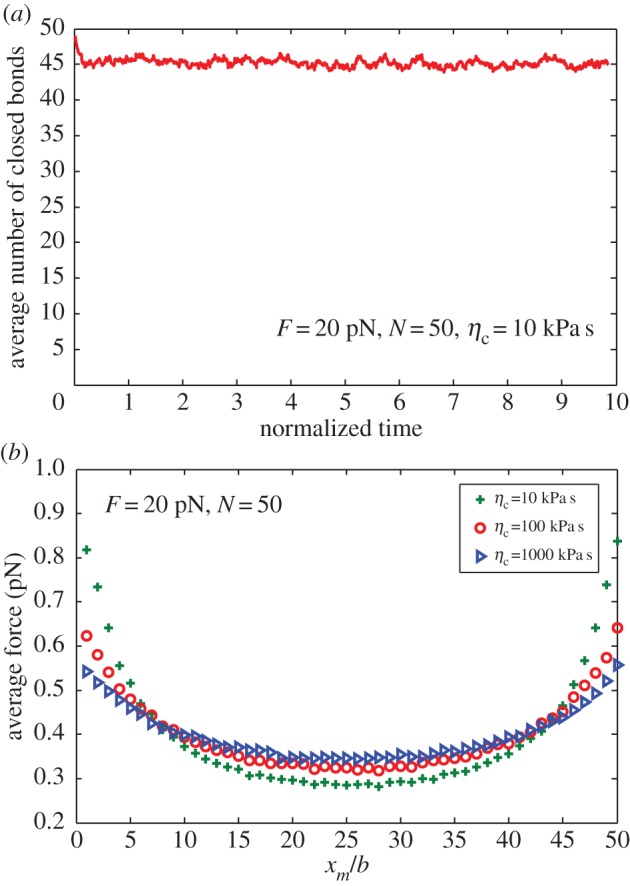
(*a*) Average number of closed bonds as a function of normalized time for loading force 20 pN, total bond number *N* = 50 and viscosity *η*_c_ = 10 kPa s. (*b*) Distribution of the average force for varying cell viscosity.

For the former, in the light of the spatial and temporal averages, figure 4*b* presents the steady-state distribution of the interfacial stress for the adhesion of viscoelastic bodies with different viscosities through a cluster of molecular bonds and under a remotely applied load. Figure 4*b* indicates that larger viscosity corresponds to a more uniformly distributed interfacial stress. From the view of individual bonds, large loading force would result in unstable binding between receptor and ligand molecules based on Bell's theory [33]. As shown in figure 4*b*, the discrete interfacial force in adhesion edges is larger than that in adhesion centre. Therefore, there is greater probability that the bonds break at edges. Once the bonds at edges break, the crack would propagate from edge to centre. On the contrary, if the force has a uniform distribution, all bonds would have stable state for adhesion.

For the latter, we note that there exist characteristic time scales for the behaviours of viscoelastic relaxation, bond dissociation and association, which can be represented by *T*_1_ = *η*_c_/*E*_c_, 1/*k*_0_ and $1/k_{\mathrm{on}}^{0}$, respectively. Competitions between these time scales determine how the cellular viscoelastic property may affect the adhesion behaviour of individual ligand–receptor bonds. For a closed bond, if the characteristic bond dissociation time 1/*k*_0_ is longer than that of the viscoelastic relaxation time *T*_1_, the deformation relaxation has no influence on bond breaking. For an open bond, when the characteristic bond association time $1/k_{\mathrm{on}}^{0}$ is longer than the characteristic relaxation time *T*_1_, the bond association is also independent of the cell viscosity, but when $1/k_{\mathrm{on}}^{0}<T_{1}$, stress relaxation may postpone the recovery of interfacial deformation from small surface separation, which makes the reforming event easier to occur.

In typical biological system, Young's modulus of cells, *E*_c_, is of the order of 10 kPa or less [74], the cell viscosity ranges from hundreds to thousands of Pa s [75], the spontaneous bond dissociation rate is in the range of 5 × 10^−6^ – 9 s^−1^ [76,77] and the spontaneous bond association rate is in the range of 1–10^3^ s^−1^ [76]. Therefore, in most cases there are always 1/*k*_0_ > *T*_1_ and $1/k_{\mathrm{on}}^{0}<T_{1}$, which implies that bond breaking is usually independent of cell viscosity, but the bond rebinding is enhanced by the cell viscosity.

**Figure 5.**
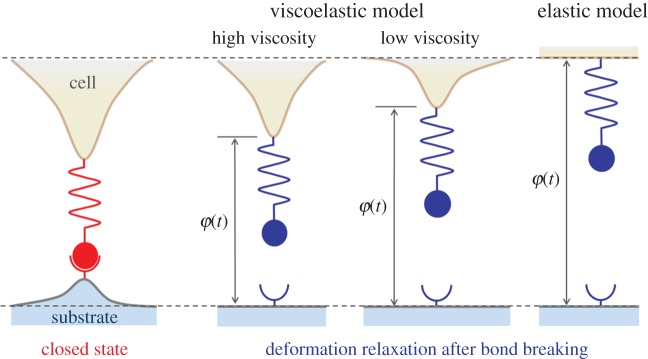
Schematic of the rebinding process of free receptor and ligand after breaking in single-bond level for viscoelastic model and elastic model of cell adhesion. The dash lines denote the cell and substrate surfaces at infinity.

In order to provide an intuitive impression of how the viscosity may increase the bond rebinding possibility, we consider an illustrative example that there is a single bond which links the cell and substrate, as shown in figure 5. If the cell has high viscosity, the recovery procedure for the cell deformation must be slower than that of the cell deformation with low viscosity after bond breaking, making a small interfacial separation between the cell and substrate. This observation indicates that the next reaction of bond rebinding at the reaction site for a cell with high viscosity occurs easier than that for the cell with low viscosity. In an extreme case, if the cell is purely elastic, deformation of the cell can be immediately recovered as long as the bond breaks. Hence, for the elastic system, large interfacial separation associates with low probability of bond rebinding.

From a biophysical point of view, cells may take advantage of their own mechanical properties for proper functioning.

**Figure 6.**
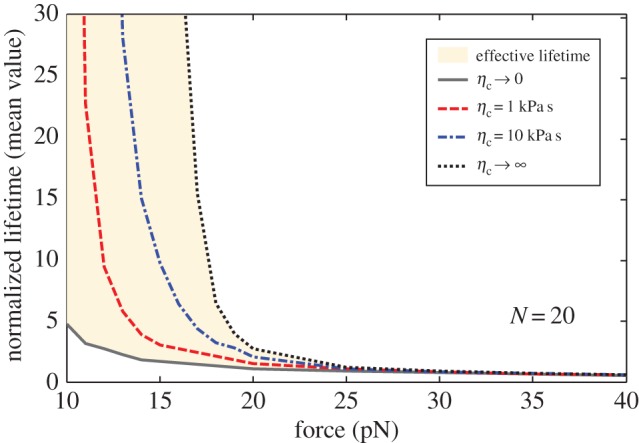
Mean normalized lifetime as a function of the applied force for varying cell viscosity and total bond number *N* = 20.

Figure 6 indicates the mean normalized lifetime for a cluster of 20 bonds as a function of applied force under different cell viscosities. Figure 6 shows that lifetime strongly depends on cell viscosity with small applied forces although this dependence becomes weak at large applied forces. This phenomenon occurs because if the applied force is large, the interfacial separation also increases. Thus, the rebinding events will rarely occur. Gupta [65] considered the adhesion of a polymorphonuclear leucocyte cell. He used a spherical membrane containing a Newtonian fluid to model the cell, and the cell was adhered to the substrate by a single molecular bond. Based on this model, Gupta [65] concluded that cellular viscoelasticity does not affect the average bond lifetime for constant-force loading, because the bond force becomes equal to the applied constant force after a transient period. This conclusion is unfortunately biased. As shown in figure 6, the rupture events of the bonds dominate and the rebinding events become rare only if the loading force is very large, and then the bond lifetime is independent of cellular viscosity. When the loading force is relatively small, the cellular viscosity will significantly influence the bond lifetime. In the work by Gupta [65], the loading force is of the order of hundreds of pNs, which can be more than 30 times larger than the typical force scale *F*_b_, leading to a very large rupture rate according to equation (2.6). By contrast, we chose the loading force in tens of PNs, which can lead to a more reasonable rupture rate. Under the present force range, the bond lifetime strongly depends on cellular viscosity, as indicated in figure 6.

Figure 6 also provides the upper (*η*_c_ → ∞) and lower (*η*_c_ → 0) bounds on the curves of lifetime versus applied force for different viscosities. Actually, if we let *η*_c_ → 0 and *η*_c_ → ∞, the creep compliance in equation (2.3) can be reduced to

3.1 $$J=\frac{3}{2E_{s}}\;\mathrm{for}\;\eta_{c}\to \infty \;\mathrm{and}\;J=\frac{3}{2E_{s}}+\frac{1}{E_{c}}\;\mathrm{for}\;\eta_{c}\to 0.$$

The above equation implies that in the case of *η*_c_ → ∞, only the substrate would deform in response to the applied force acting on the interface. For *η*_c_ → 0, both the substrate and cell would deform. Previous experimental and theoretical studies [23,24,45] demonstrated that substrate elasticity can regulate cell adhesion. In this study, we broaden this conclusion by determining that the cellular viscosity can also significantly affect cell adhesion.

### 3.2. Effects of cellular viscosity on the dynamic strength of bond cluster

Our model can also be expanded to predict the dynamic adhesion between the cell and substrate via receptor–ligand bonds. We consider the displacement controlled loading by setting interfacial separation *h* as *h* = *λt*, where *λ* is the loading rate. In this case, consultant interfacial force *F* is no longer constant.

**Figure 7.**
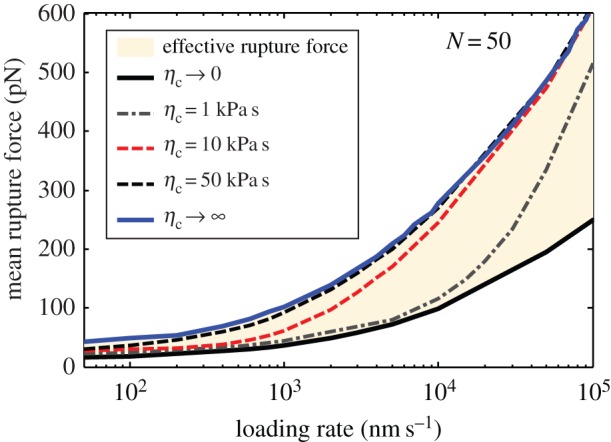
Mean rupture force as a function of loading rate under displacement loading for varying cell viscosity and total bond number *N* = 50.

The mean rupture force of a bond cluster with *N* = 50 is numerically determined and plotted in figure 7 as a function of the loading rate under different cell viscosities. The figure indicates that, if the loading rate is low, the mean rupture force exhibits an asymptotical strength limit and the cellular viscosity only slightly affects the bond strength. This phenomenon was also observed by Li & Ji [78,79] in studying the stretch of a single molecular bond. The asymptotical bond strength results from the reaction equilibrium between the receptor and ligand, as most recently revealed by Li *et al*. [80]. Furthermore, if the loading rate is large, the dynamic strength of the bond cluster increases with loading rate and the cellular viscosity slightly affects adhesion strength.

If the loading rate is intermediate large, as shown in figure 7, cellular viscosity can significantly enhance the dynamic strength of the bond cluster. This loading rate range that leads to viscosity-dependent bond strength is a result of the competition between time scales of bond formation, loading rate and creep. When the cell separates from the substrate under a low loading rate, interfacial deformation has sufficient time to recover so that the cellular viscosity does not affect mean rupture force. In the case of large loading rate, the dynamic strength is dominated by the fast increase of interfacial separation between the cell and substrate, implying that the rebinding events rarely occur after bond-breaking. Thus, the rupture force of the bond cluster depends on the cellular viscosity only in the intermediate range of loading rate.

Moreover, figure 7 shows the upper (*η*_c_ → ∞) and lower (*η*_c_ → 0) bounds on the curves of effective mean rupture force versus loading rate for different viscosities. The figure indicates that the rupture force of the bond cluster does not change with viscosity when the viscosity exceeds 50 kPa s. Interestingly, in nature, the value of viscosity for most cells is in the range of tens to thousands of Pa s [81–83], implying that cells may adapt their own viscosity by cytoskeleton remodelling to control adhesion.

### 3.3. Effects of cellular viscosity on the window of stable adhesion

**Figure 8.**
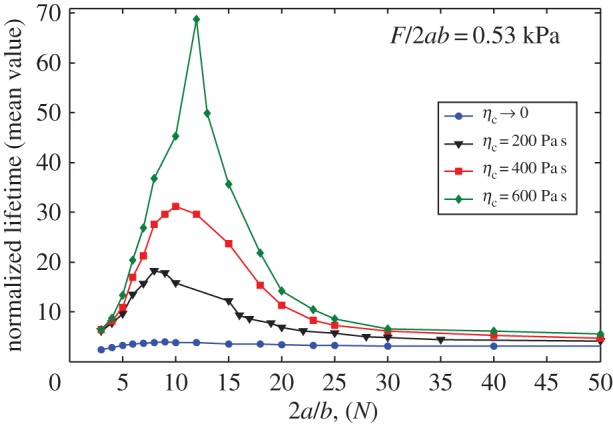
Mean value of the normalized lifetime as a function of cluster size for varying cell viscosity under a fixed load of 0.53 kPa.

Normalized lifetime of the adhesion cluster is shown in figure 8 as a function of cluster size 2*a*/*b* for constant stress *F*/2*ab* = 0.53 kPa and different cell viscosities. Evidently, a size-window exists for the stable adhesion of the molecular bond cluster. These size effects on cell adhesion were demonstrated based on the model that disregards the effects of viscosity [40,45,50]. By contrast, in this study, we found that cell viscosity can increase the lifetime and also broaden the size of the stable window, indicating that the cell may use its own viscous property other than or together with elasticity property to maintain the stable adhesion in a large range of cluster sizes. Why there exists such a size-window: if bond cluster size is small, since few bonds are involved in maintaining stable adhesion, the stochastic breaking and reforming of molecular bonds would lead to a short lifetime. For very large bond cluster size, the stress concentration results in the cluster breaking from edge to centre and also eventually makes a short lifetime. Therefore, there will be an optimal cluster size that maximizes the adhesion strength.

## 4. Conclusion

In this research, we developed an idealized viscoelastic–stochastic model of two elastic and viscoelastic bodies connected by a receptor–ligand bond cluster. Based on this model, we have performed Monte Carlo simulations to determine the role of cellular viscosity in biological behaviour of cell adhesion, such as lifetime, dynamic strength and stable window, by coupling the continuum deformation of the viscoelastic cell and elastic substrate and the stochastic behaviour of molecular bonds. The main conclusions are summarized as follows. (1) We broadened the concept of the stress concentration index given by Qian *et al*. [45] to include the effect of stress relaxation. Based on such a dynamic stress concentration index, we pointed out that cellular viscosity can reduce stress concentration at the adhesion domain. (2) We sufficiently demonstrated that the time-dependent recovery process of viscoelastic deformation increases the probability of rebinding for free receptors and ligands at binding sites. Thus, cellular viscosity can generally prolong the lifetime of an adhesion cluster. However, once the loading force is large, the effect of cellular viscosity on the bond lifetime becomes negligible. That is why Gupta [65] did not observe the effect of cellular viscosity on bond lifetime under constant-force loading. (3) Compared with the results in the previous study [64], our analysis clearly shows that cellular viscosity enhances rather than reduces the adhesion strength of a cluster of molecular bonds under a displacement controlled dynamic loading. (4) Cellular viscosity also plays an important role in controlling the size-window for stable adhesion of a molecular bond cluster. Cellular viscosity can effectively broaden the stable size-window and prolong cluster lifetime but only for clusters within this size-window. This finding indicates that cellular viscosity can exert the same effect on the adhesion stabilization as substrate stiffness [45].

*In vitro* experiments on cells can be designed to verify the proposed theory, for example, some nanoparticles and drugs can be used to change the viscosity of the cells, and such a viscosity change can be reflected through the change in their adhesion states as revealed by the present theory.

Overall, the results based on this model, although overly simplified in this study, seem to support a novel view on understanding the role of cell viscosity in cell adhesion.

## Appendix A. Dynamic interfacial traction

In this study, the cell and substrate are considered as linear incompressible viscoelastic and elastic half spaces, respectively. For the cell, stresses and strains at any points are related as follows [84]:

A 1 $$e_{ij}^{c}(t)=J_{c}(t)*dS_{ij}^{c}(t),$$

where ‘*’ represents convolution operator, the subscript and superscript ‘c’ represents ‘cell’, *J*_c_(*t*) stands for the shear creep modulus of the viscoelastic body, $e_{ij}^{c}$ and $S_{ij}^{c}$ are deviatoric components of the strain and stress tensors, respectively, which are given by

A 2 $$e_{ij}^{c}=\varepsilon_{ij}^{c}-\frac{1}{3}\delta_{ij}\varepsilon_{ii}^{c}\;\mathrm{and}\;S_{ij}^{c}=\sigma_{ij}^{c}-\frac{1}{3}\delta_{ij}\sigma_{ii}^{c},$$

where *δ_ij_* is the Kronecker delta, $\varepsilon_{ij}^{c}$ and $\sigma_{ij}^{c}$ are components of the strain and stress tensors, respectively, and $\varepsilon_{ii}^{c}$ and $\sigma_{ii}^{c}$ are the volume strain and volume stress, respectively. For incompressible material, one has

A 3 $$e_{ij}^{c}=\varepsilon_{ij}^{c}\;\mathrm{and}\;S_{ij}^{c}=\sigma_{ij}^{c}.$$

The stress equilibrium equation and the strain–displacement relationship are then expressed as

A 4 $$\sigma_{ij,j}^{c}=0\;\mathrm{and}\;\varepsilon_{ij}^{c}=\frac{1}{2}(u_{i,j}^{c}+u_{j,i}^{c}),$$

where $u_{i}^{c}$ are components of the displacement vector. The stress and displacement should follow the boundary conditions as

A 5 $$\sigma_{ij}^{c}n_{j}=P_{i},\;\mathrm{on}\;S_{P}$$

and

A 6 $$u_{i}^{c}=D_{i},\;\mathrm{on}\;S_{D},$$

where *n_j_* are the components of the unit outward normal to the boundary, *S_P_* and *S_D_*, *P_i_* are the tractions prescribed on boundary *S_P_*, and *D_i_* are the displacements prescribed on boundary *S_D_*.

After inserting equation (A 3) into equation (A 1), the Laplace transforms of equations (A 1), (A 4)–(A 6) are obtained as [84]

A 7 $$\begin{array}{rl} {\hat{\varepsilon }}_{ij}^{c} & =s{\hat{J}}_{c}{\hat{\sigma }}_{ij}^{c}, \end{array}$$

A 8 $$\begin{array}{rl} {\hat{\sigma }}_{ij,j}^{c} & =0, \end{array}$$

A 9 $$\begin{array}{rl} {\hat{\varepsilon }}_{ij}^{c} & =\frac{1}{2}({\hat{u}}_{i,j}^{c}+{\hat{u}}_{j,i}^{c}), \end{array}$$

A 10 $$\begin{array}{rl} {\hat{\sigma }}_{ij}^{c}n_{j} & ={\hat{P}}_{i},\;\mathrm{on}\;S_{P} \end{array}$$

A 11 $$\begin{array}{rl} \mathrm{and}\;{\hat{u}}_{i}^{c} & ={\hat{D}}_{i},\;\mathrm{on}\;S_{D}. \end{array}$$

Obviously, it can be seen from equations (A 7)–(A 11) that linear viscoelastic deformation is transformed into elastic deformation in the Laplace transformed state, which agrees with the so-called correspondence principle [84,85].

Specifically, the interfacial traction can be obtained as

A 12 $$\gamma (x,t)=\rho_{\mathrm{LR}}k_{\mathrm{LR}}\rho (x,t)\xi (x,t),$$

where *ρ*_LR_ is the bond density when all bonds are closed, *ρ*(*x*, *t*) is the normalized density of closed bonds at position *x* and time *t* with respect to *ρ*_LR_, *k*_LR_ denotes the stiffness of the bond and *ξ*(*x*, *t*) is the elastic extension of the bond at position *x* and time *t*.

For such linear problem, in Laplace-transform space, the surface displacement gradient $\partial {\hat{w}}_{c}(x,s)/\partial x$ of the viscoelastic body can be related to the interfacial traction $\hat{\gamma }(x,s)$ through Green's function

A 13 $$\frac{\partial {\hat{w}}_{c}(x,s)}{\partial x}=-\frac{1}{\pi }\int_{-a}^{a}\frac{s{\hat{J}}_{c}(s)\hat{\gamma }(x,s)}{x-z}\;dz,$$

where *a* is half size of the adhesion patch, $\hat{\gamma }(x,s)$ and $\hat{w}(x,s)$ correspond to the Laplace transform of *γ*(*x*,*t*) and *w*(*x*, *t*) with respect to time *t*.

Applying inverse Laplace transform to equation (A 13) yields

A 14 $$\frac{\partial w_{c}(x,t)}{\partial x}=-\frac{1}{\pi }\int_{-a}^{a}\frac{1}{x-z}\int_{0}^{t}J_{c}(t-\tau )\frac{d\gamma (z,\tau )}{d\tau }\;d\tau dz.$$

Meanwhile, the deformation of the elastic substrate would be induced by the interfacial traction *γ*(*x*,*t*). The surface displacement gradient $\partial w_{s}(x,t)/\partial x$ of the elastic body has a relation with the interfacial traction *γ*(*x*, *t*) through Green's function as [86]

A 15 $$\frac{\partial w_{s}^{\;}}{\partial x}=-\frac{2(1-\nu_{s}^{2})}{\pi E_{s}}\int_{-a}^{a}\frac{\gamma (z,t)}{x-z}\;dz,$$

where superscript ‘s’ represents the ‘substrate’, *E*_s_ and *v*_s_ are Young's modulus and Poisson's ratio of the substrate, respectively. For incompressible material, *v*_s_ equals 0.5.

By combining equations (A 14) and (A 15), the relative normal surface deformation gradient of the two bodies can be related to the interfacial traction as

A 16 $$\frac{\partial w(x,t)}{\partial x}=\frac{\partial w_{c}(x,t)}{\partial x}+\frac{\partial w_{s}(x,t)}{\partial x}=-\frac{1}{\pi }\int_{-a}^{a}\frac{1}{x-z}\int_{0}^{t}J(t-\tau )\frac{d\gamma (z,\tau )}{d\tau }\;d\tau dz,$$

where the relative normal surface deformation *w*(*x*, *t*) is denoted as the sum of surface deformations of the elastic and viscoelastic half spaces at position *x* and time *t*, i.e. *w*(*x*, *t*) = *w*_c_(*x*, *t*) + *w*_s_(*x*, *t*), as shown in figure 2. If the cell is considered as the Kelvin–Voigt solid with Young's modulus *E*_c_ and viscosity coefficient *η*_c_, then the combined creep compliance *J*(*t*) can be expressed as

A 17 $$J(t)=J_{s}(t)+J_{c}(t)=\frac{3}{2E_{s}}+\frac{1}{E_{c}}\left(1-\exp\left(-\frac{t}{T_{1}}\right)\right),$$

where *J*_s_(*t*) = 3/2*E*_s_, *J*_c_(*t*) = (1 − exp(−*t*/*T*_1_))/*E*_c_ and *T*_1_ = *η*_c_/*E*_c_ represent the characteristic time scale of relaxation.

## Appendix B. Association rate of receptor–ligand bonds

The reaction between receptor and ligand molecules is mediated by the surface separation *φ* as shown in figure 2. At a given separation *φ*, the truncated harmonic potential for a free receptor molecules has the form

B 1 $$U(y)=\frac{1}{2}k_{\mathrm{LR}}y^{2}\;\mathrm{and}\;y\in [-l_{b},\varphi -l_{b}],$$

where *y* is the possible displacement of the receptor molecule as displayed in figure 2. Consider that the receptor displacement follows the Boltzmann distribution, the probability density function for the receptor with displacement *y* is

B 2 $$P(y)=\frac{1}{Z}\exp\left(-\frac{U(y)}{k_{B}T}\right)=\frac{1}{Z}\exp\left(-\frac{k_{\mathrm{LR}}y^{2}}{2k_{B}T}\right)\;\mathrm{and}\;y\in [-l_{b},\varphi -l_{b}],$$

where *Z* is the partition function, which can be given by solving the normalization equation $\int_{-l_{b}}^{\varphi -l_{b}}P(y)\;dy=1$ as

B 3 $$Z=\sqrt{\frac{\pi k_{B}T}{2k_{\mathrm{LR}}}}\left(\mathrm{erf}\left((\varphi -l_{b})\sqrt{\frac{k_{\mathrm{LR}}}{2k_{B}T}}\right)+\mathrm{erf}\left(l_{b}\sqrt{\frac{k_{\mathrm{LR}}}{2k_{B}T}}\right)\right),$$

where erf(.) is the error function. Hence, the probability that the receptor falls into a reacting radius *l*_bind_ around the binding site is

B 4 $$p=\int_{\varphi -l_{b}-l_{\mathrm{bind}}}^{\varphi -l_{b}}P(y)dy=\frac{l_{\mathrm{bind}}}{Z}\exp\left(-\frac{k_{\mathrm{LR}}{\left(\varphi -l_{b}\right)}^{2}}{2k_{B}T}\right).$$

Therefore, the bond association rate is given as [45,68,69]

B 5 $$k_{\mathrm{on}}=k_{\mathrm{on}}^{0}\frac{l_{\mathrm{bind}}}{Z}\exp\left(-\frac{k_{\mathrm{LR}}{\left(\varphi -l_{b}\right)}^{2}}{2k_{B}T}\right),$$

where $k_{\mathrm{on}}^{0}$ is the spontaneous reaction rate between the receptor and ligand.

## Appendix C. Forces and displacements for discrete bonds

For the cell–substrate adhesion system as shown in figure 1, we consider the stochastic process of the cluster of discretely distributed receptor–ligand bonds. We use theory of continuum mechanics to determine the forces and displacements at bond sites. Specifically, for a given bond location *x_m_* within the adhesion patch, we will determine relative interfacial displacement *w_mn_* induced by a force *F_n_* acting on another bond location, *x_n_* (*m* ≠ *n*).

In appendix A, we have derived

C 1 $$\frac{\partial w(x,t)}{\partial x}=-\frac{1}{\pi }\int_{-a}^{a}\frac{1}{x-z}\int_{0}^{t}J(t-\tau )\frac{d\gamma (z,\tau )}{d\tau }\;d\tau \;dz.$$

Integrating both sides of equation (C 1) yields

C 2 $$w(x,t)=\frac{1}{\pi }\int_{-a}^{a}\ln\left|\frac{x_{\infty }-z}{x-z}\right|\int_{0}^{t}J(t-\tau )\frac{d\gamma (z,\tau )}{d\tau }\;d\tau \;dz,$$

where we have assumed *w*(*x*_∞_,*t*) = 0 when *x*_∞_ is sufficiently large.

Assuming that only one bond force, *F_n_*(*t*), is applied at bond location *x_n_*, so that

C 3 $$\gamma (z,\tau )=\frac{F_{n}(t)}{b}\delta (z-x_{n}).$$

Substituting equation (C 3) into equation (C 2) yields

C 4 $$w(x,t)=\frac{1}{\pi b}\ln\left|\frac{x_{\infty }-x_{n}}{x-x_{n}}\right|\int_{0}^{t}J(t-\tau )\frac{dF_{n}}{d\tau }\;d\tau .$$

Applying *x* = *x_m_*, *m* ≠ *n*, to equation (C 4), we have

C 5 $$w_{mn}=w(x_{m},t)=\frac{1}{\pi b}\ln\left|\frac{x_{\infty }-x_{n}}{x_{m}-x_{n}}\right|\int_{0}^{t}J(t-\tau )\frac{dF_{n}}{d\tau }\;d\tau ,$$

which represents the displacement at position *x_m_* to be induced by bond force *F_n_* at position *x_n_*.

On the other hand, in order to derive the displacement at position *x_n_* to be induced by the bond force *F_n_* at the same position, we replace the concentrate force *F_n_* by an equivalent uniform pressure with half-width *a*_0_. In this case, equation (C 2) gives

C 6 $$\begin{array}{rl} w(x_{n},t) & =\frac{1}{2a_{0}b\pi }\int_{x_{n}-a_{0}}^{x_{n}+a_{0}}\ln\left|\frac{x_{\infty }-z}{x_{n}-z}\right|\int_{0}^{t}J(t-\tau )\frac{dF_{n}(\tau )}{d\tau }\;d\tau \;dz\\ & =C\int_{0}^{t}J(t-\tau )\frac{dF_{n}(\tau )}{d\tau }\;d\tau , \end{array}$$

where

C 7 $$C\approx \frac{1}{2a_{0}b\pi }\int_{-a_{0}}^{a_{0}}\ln\left|\frac{x_{\infty }-z}{z}\right|\;dz=\frac{1}{a_{0}b\pi }\left(1+a_{0}\ln\frac{x_{\infty }^{\;}}{a_{0}}\right),$$

and $x_{\infty }\gg x_{n}$, $x_{\infty }\gg a_{0}$ have been considered. Therefore, the self-displacement at *x_n_* induced by the force *F_n_* can be given by

C 8 $$w_{nn}=C\int_{0}^{t}J(t-\tau )\frac{dF_{n}(\tau )}{d\tau }\;d\tau .$$

Then, for a given time *t*, the total relative interfacial displacement at location *x_m_* becomes

C 9 $$\begin{array}{rl} w(x_{m}) & =w_{mm}+\sum_{n=1,n\neq m}^{N}w_{mn}\\ & =C\int_{0}^{t}J(t-\tau )\frac{dF_{m}(\tau )}{d\tau }\;d\tau +\sum_{n=1,n\neq m}^{N}\left[\frac{\ln|\left(x_{\infty }-x_{n}\right)/(x_{m}-x_{n})|}{\pi b}\int_{0}^{t}J(t-\tau )\frac{dF_{n}(\tau )}{d\tau }\;d\tau \right], \end{array}$$

where *N* is the total number of receptor–ligand bonds.

The total force acting on each closed bond should balance the applied external force as

C 10 $$\sum_{n=1}^{N}F_{n}=F.$$

For open-bond location *x_n_*, as shown in figure 2 the separation between two half spaces, *φ,* at position, *x_n_*, presents the form

C 11 $$\varphi (x_{n},t)=W(t)-w(x_{n},t),$$

and the exerted force is zero, *F_n_* = 0. For closed-bond location *x_m_*, the geometrical relationship is

C 12 $$w(x_{m},t)+\xi (x_{m},t)+l_{b}+l_{\mathrm{bind}}=W(t).$$

Combining equations (C 10)–(C 12) gives the exerting force on each bond and the separations for open bonds.
